# Development of a Comprehensive Sequencing Assay for Inherited Cardiac Condition Genes

**DOI:** 10.1007/s12265-016-9673-5

**Published:** 2016-02-17

**Authors:** Chee Jian Pua, Jaydutt Bhalshankar, Kui Miao, Roddy Walsh, Shibu John, Shi Qi Lim, Kingsley Chow, Rachel Buchan, Bee Yong Soh, Pei Min Lio, Jaclyn Lim, Sebastian Schafer, Jing Quan Lim, Patrick Tan, Nicola Whiffin, Paul J. Barton, James S. Ware, Stuart A. Cook

**Affiliations:** National Heart Research Institute Singapore, National Heart Centre Singapore, 168752 Singapore, Singapore; Division of Cardiovascular & Metabolic Disorders, Duke-National University of Singapore, 169857 Singapore, Singapore; NIHR Cardiovascular Biomedical Research Unit, Royal Brompton and Harefield NHS Foundation Trust and Imperial College London, London, SW3 6NP UK; National Heart and Lung Institute, Imperial College London, London, SW3 6LY UK; Division of Medical Sciences, National Cancer Centre Singapore, 169610 Singapore, Singapore; Division of Cancer and Stem Cell Biology, Duke-National University of Singapore, 169857 Singapore, Singapore; SingHealth/Duke-NUS Precision Medicine Institute, National Heart Centre Singapore, 168752 Singapore, Singapore; MRC Clinical Sciences Centre, Imperial College London, London, W12 0NN UK

**Keywords:** Inherited cardiac conditions, Targeted sequencing, Whole exome sequencing, Whole genome sequencing, Genetics, Diagnostics

## Abstract

Inherited cardiac conditions (ICCs) are characterised by marked genetic and allelic heterogeneity and require extensive sequencing for genetic characterisation. We iteratively optimised a targeted gene capture panel for ICCs that includes disease-causing, putatively pathogenic, research and phenocopy genes (*n* = 174 genes). We achieved high coverage of the target region on both MiSeq (>99.8 % at ≥20× read depth, *n* = 12) and NextSeq (>99.9 % at ≥20×, *n* = 48) platforms with 100 % sensitivity and precision for single nucleotide variants and indels across the protein-coding target on the MiSeq. In the final assay, 40 out of 43 established ICC genes informative in clinical practice achieved complete coverage (100 % at ≥20×). By comparison, whole exome sequencing (WES; ∼80×), deep WES (∼500×) and whole genome sequencing (WGS; ∼70×) had poorer performance (88.1, 99.2 and 99.3 % respectively at ≥20×) across the ICC target. The assay described here delivers highly accurate and affordable sequencing of ICC genes, complemented by accessible cloud-based computation and informatics. See Editorial in this issue (DOI: 10.1007/s12265-015-9667-8).

**Electronic supplementary material:**

The online version of this article (doi:10.1007/s12265-016-9673-5) contains supplementary material, which is available to authorized users.

## Introduction

Inherited cardiac conditions (ICCs) are diseases of the heart and circulation with a combined prevalence of ∼1 %. ICCs include inherited arrhythmia syndromes, cardiomyopathies, aortopathies and hyperlipidaemias [1–4]. They most commonly exhibit autosomal dominant inheritance, though with highly variable expressivity and penetrance. Sequencing of ICC genes can be performed to confirm an ICC diagnosis, inform patient management/cascade screening and be useful for molecular autopsy in the case of sudden unexplained death [5].

Until recently, Sanger sequencing was used for ICC gene sequencing for both clinical and research applications, but this technique has limited throughput and is prohibitively expensive for large genes/large numbers of genes [6]. Next-generation sequencing (NGS) reduces the cost and increases throughput of gene sequencing and can now be performed on inexpensive bench-top NGS platforms [7]. Bench-top sequencers have the advantages of low capital cost, compact footprint and a simplified workflow compared to high-throughput sequencers, yet still meet the sequencing needs of individual research and clinical laboratories [8].

Whole exome sequencing (WES) and targeted sequencing have been developed as alternatives to whole genome sequencing (WGS). These approaches have reduced sequencing costs, turnaround times, data storage needs and informatics burdens compared to WGS. There are many approaches to enrich for target sequences that use varying DNA preparation and capture methods that can be in solution, solid-phase or PCR-based [9]. In solution, WES is a popular off-the-shelf choice, as assays have been designed to capture all human genes. However, WES often results in uneven coverage across and between genes and can particularly struggle with GC-rich regions such as first exons [10]. The interpretation of incidental variants, as suggested by American College of Medical Genetics and Genomics (ACMG), is also a potential issue for WES where variants unrelated to the patient’s referral condition may be detected [11]. Augmented WES assays containing additional probes targeting disease genes have been developed, but average assay performance remains suboptimal (∼90 %, ≥20×) and WGS may perform better [12].

Targeted sequencing of gene panels is an alternative to WES and has been widely used in research and is increasingly applied in clinical settings [13]. In the ICC setting, small gene panels have been used for specific ICCs, including long QT syndrome (LQTS), hypertrophic cardiomyopathy (HCM), dilated cardiomyopathy (DCM) and arrhythmogenic right ventricular cardiomyopathy (ARVC) [14–16]. Multiple workflows and bioinformatics pipelines are needed to run these various ICC gene panels, and gene coverage is such that Sanger sequencing ‘fill in’ is needed, which has very major manpower implications.

Here, we describe the development of a new gene panel for ICCs, which provides a comprehensive, single workflow assay with high levels of coverage across all ICC genes for use in research and ultimately clinical settings. The performance of the panel was iteratively improved by probe design, across sequencing platforms and by sequencing chemistry refinement. Assay performance was assessed in detail compared to WES and WGS using local and cloud-based informatics pipelines.

## Materials and Methods

### Subject Specimens

Subjects (*n* = 348) were recruited from National Heart Centre Singapore and via advertisement at the MRC Clinical Sciences Centre, Imperial College London. Samples for WGS (*n* = 8) were obtained from National Cancer Centre Singapore, National University Hospital Singapore. All participants gave written informed consent, and study protocols were approved by the local institutional ethics committees and carried out in accordance with local Tissue Acts, as appropriate. Genomic DNA was extracted from blood using Prepito DNA Blood 600 kit (Perkin Elmer, MA) (targeted sequencing), EZ1 DSP DNA blood 48 kit (Qiagen, Netherlands) (WES) or QIAsymphony DNA kit (Qiagen, Netherlands) (WGS) following manufacturer’s protocols. Quality and quantity of extracted DNA were assessed by an ultraviolet-visible spectrophotometer.

### Targeted Enrichment

An initial ICC gene panel targeting 169 ICC genes (ICCv1, target region = 1.47 Mb; including 3′ and 5′UTRs) and an iterated version targeting 174 genes (ICCv2, target region = 0.57 Mb; protein coding ± 40 bp buffer) were designed using Illumina Design Studio (San Diego, CA). Genes were chosen on the basis of reported associations of disease-causing variants with relevant ICCs which were identified in the Human Gene Mutation Database (HGMD) Professional version 2014.1, followed by manual curation and addition of further genes of research interest by a team of cardiologists and clinical geneticists (Table S1). ICCv2 BED file with targets and genomic coordinates are provided in Table S2. The 169 ICC genes consistently represented in all sequence capture panels were assessed for the purposes of this study. Libraries were prepared using Nextera Rapid Capture Enrichment kits according to the manufacturer’s protocols.

### Targeted, Whole Exome and Whole Genome Sequencing

Targeted sequencing: Pooled libraries (*n* samples = 6–48) prepared using the ICC panel were sequenced on the Illumina MiSeq (v2 kit; *n* = 108) or NextSeq 500 (Mid Output v2 kit, *n* = 144) benchtop sequencers using paired-end, 150 bp reads. WES: 96 samples underwent WES using the Nextera Rapid Capture Exome kit according to the manufacturer’s instructions. Each pool (*n* = 12) was sequenced on a single lane of the HiSeq 2500 (SBS v4 kit, 125 bp paired-end (PE) reads, yielding ≥4 GB of raw data per sample, ∼mean read depth of 80× and >80 % of bases at >10×). Deep WES: Six out of 96 WES samples were randomly selected, and all reads were combined to obtain sequencing depth equivalent to that acquired by ICC panel sequencing in typical use (∼43 GB of raw data per sample, ∼mean read depth of 500×). WGS: Eight samples were prepared using the TruSeq Nano DNA kit according to the manufacturer’s instructions. Each sample was sequenced on two lanes of the HiSeq X (v2.5 kit, 150 bp PE reads, yielding ∼200 GB of raw data per sample, ∼mean read depth of 70×).

### Sequence Alignment and Variant Calling

Raw sequencing data (.bcl files) were demultiplexed into individual FastQ read files with Illumina’s bcl2fastq v2.16.0.10 based on unique index pairs. Low quality (*Q* < 20) reads/bases were trimmed using Trimmomatic v0.3220.4 [17], and read quality was assessed using FastQC v0.10.1 [18]. High-quality reads were mapped to UCSC GRCh37/hg19 reference genome using Burrows-Wheeler Aligner (BWA) v0.7.10 [19]. Picard v1.119 and The Genome Analysis Toolkit (GATK) v3.3 [20] were used to mark duplicate reads, realign locally around indels and recalibrate base quality scores according to best practices. Alignment summary metrics and coverage and callability metrics were generated using Picard v1.119, SAMtools v1.1 [21], Bedtools v2.17 [22] and in-house Perl/Shell scripts. A base was considered ‘callable’ if sequenced with minimum read depth = 20×, base quality ≥ 20 and mapping quality ≥ 20. GATKv3.3 HaplotypeCaller and UnifiedGenotyper were used to call variants from reads mapped with quality ≥ 20. Variants were annotated with Ensembl Variation database v75_37 [23] and HGMD Professional version 2014.1 [24]. Among all 252 samples sequenced using the ICC panel, there were ten outliers (defined as total number of reads per sample greater than third quartile +1.5 inter-quartile range (IQR) or below first quartile −1.5 IQR), which were excluded from the analysis. In addition, 11 WES samples with <80 % of bases at >10× were excluded from analysis. Pathogenic or likely pathogenic variants (*n* = 26) identified using the ICC panel in a research cohort (*n* = 35) were subjected to Sanger sequencing. In addition to our in-house pipeline described above, a subset of samples (*n* = 65) were also analysed using the BWA Enrichment v2.1 and Isaac Enrichment v2.1 available in Illumina’s cloud genomics platform (https://basespace.illumina.com), and variant calling data was compared to the in-house GATK HaplotypeCaller pipeline (Table S3) [25, 26].

### Sensitivity and Precision of Variant Calling

Sensitivity and precision of variant calling of the ICC panel were assessed using the NA12878 reference sample. High confidence regions and the associated variant calls were downloaded from Genome in a Bottle (GIAB) (ftp://ftp-trace.ncbi.nlm.nih.gov/giab/ftp/release/NA12878_HG001/NISTv2.19/) [27] and compared to variant calls from ICC panel sequencing on both the MiSeq and NextSeq platforms. Variant calls were defined as true positive (TP) for those identified from panel sequencing and by GIAB, false positive (FP) for those identified as reference by GIAB but as variant in panel sequencing, false negative (FN) for variants identified by GIAB but not by panel sequencing and true negative (TN) for bases identified as reference in both the GIAB call set and panel sequencing. Sensitivity was calculated as TP / (TP + FN) and precision as TP / (TP + FP). Finally, we calculated the Matthews correlation coefficient (MCC), an alternative accuracy measure that takes into account unbalanced data, using the following equation: (TP × TN) − (FP × FN) / √[(TP + FP)(TP + FN)(TN + FP)(TN + FN)].

## Results

### ICC Panel: Optimisation and Performance

The performance across the iteratively improved ICC gene panels was compared using a callability metric (minimum read depth = 20×, base quality ≥ 20 and mapping quality ≥ 20) that defines adequate coverage for robust variant calling. Four methods were compared (Table S4). First, ICCv1 (169 genes, 1.47 Mb target) was sequenced at standard multiplex (method 1; M1). The mean callability of all genes using ICCv1 was proportional to the number of mapped reads per sample at low depth but saturated at ∼4 M mapped reads per sample. The low overall performance of M1 (∼94 % target, mean read depth > 20×) reflected low capture efficiency of specific gene regions as opposed to a global effect. In an attempt to improve assay performance, we included fewer samples per run (*n* = 6, method 2 (M2)) resulting in better performance (99.8 % target, ≥20×) but at a greater cost (Table S4). Overall, the performance of the M1 and M2 assays were suboptimal and are not referred to further.

We then made a major iteration of the target capture assay in ICCv2 by reducing the target (size = 0.57 Mb) to the coding DNA sequence only and by modifying the baits targeting poorly captured regions. The ICCv2 assay consisted of 174 genes, of which 169 genes were shared with the ICCv1 panel (169 genes, size = 0.56 Mb) and are considered in the comparisons presented here (Table S1). Libraries prepared with ICCv2 were sequenced either on the MiSeq (method 3, (M3)) or the NextSeq 500 (method 4, (M4)). Both M3 and M4 achieved major improvements in overall performance when compared to M1 and M2, in additional to a reduced sequencing cost per sample (Table S4).

### MiSeq Versus NextSeq 500 Sequencing

The maximal data output of the MiSeq was 6.6 GB (up to 42.1 million paired-end reads passing filters), while the NextSeq 500 generated up to 65 GB of data (up to 420 million paired-end reads passing filters). Limited duplicate reads (∼15 %) were observed with MiSeq runs, while a twofold increased duplicate reads were found (∼30 %) with NextSeq 500 runs, likely reflecting limited library complexity (fragment start sites and insert sizes of PE reads). Using ICCv2 and the NextSeq platform, the coverage of well-characterised, disease-causing genes across the major ICC disease classes was 100 % for 40 out of the 43 genes (Table 1). Small recurrent gaps in gene coverage occurred in three important genes: TGFBR1 (97 bp, exon 1), MYH7 (160 bp, exon 27) and TTN (72 to 90 bp, 19 exons (168–252)) (Table 1).

**Table 1:** ICC disease genes (*n* = 43) categorised by primary disease association and regions not covered at 20× read depth using ICCv2 and NextSeq 500 sequencing

Cardiac diseases	Core genes	Gene description	Mean callability at 20× coverage (95 % CI)	Base pairs (bp) with <20× read depth
Aortopathies	ACTA2	Actin, alpha 2, smooth muscle, aorta	100 (100–100)	0
	COL3A1	Collagen, type III, alpha 1	100 (100–100)	0
	FBN1	Fibrillin 1	100 (100–100)	0
	MYH11	Myosin, heavy chain 11, smooth muscle	100 (100–100)	0
	TGFB2	Transforming growth factor, beta 2	100 (100–100)	0
	TGFBR1	Transforming growth factor, beta receptor 1	98.0 (97.8–98.2)	97
	TGFBR2	Transforming growth factor, beta receptor II (70/80 kda)	100 (100–100)	0
Arrhythmogenic right ventricular cardiomyopathy (ARVC)	DSC2	Desmocollin 2	100 (100–100)	0
	DSG2	Desmoglein 2	100 (100–100)	0
	DSP	Desmoplakin	100 (100–100)	0
	JUP	Junction plakoglobin	100 (100–100)	0
	PKP2	Plakophilin 2	100 (100–100)	0
Brugada syndrome (BrS)	SCN5A	Sodium channel, voltage-gated, type V, alpha subunit	100 (100–100)	0
Catecholaminergic polymorphic ventricular tachycardia (CPVT)	CASQ2	Calsequestrin 2 (cardiac muscle)	100 (100–100)	0
	RYR2	Ryanodine receptor 2 (cardiac)	100 (100–100)	0
Dilated cardiomyopathy (DCM)	DES	Desmin	100 (100–100)	0
	LMNA	Lamin A/C	100 (100–100)	0
	MYBPC3	Myosin-binding protein C, cardiac	100 (100–100)	0
	MYH7	Myosin, heavy chain 7, cardiac muscle, beta	100 (99.9–100)	160
	RBM20	RNA binding motif protein 20	100 (100–100)	0
	TNNI3	Troponin I type 3 (cardiac)	100 (100–100)	0
	TNNT2	Troponin T type 2 (cardiac)	100 (100–100)	0
	TPM1	Tropomyosin 1 (alpha)	100 (100–100)	0
	TTN	Titin	99.7 (99.7–99.8)	1569
Familial hypercholesterolaemia (FH)	APOB	Apolipoprotein B (including Ag(x) antigen)	100 (100–100)	0
	LDLR	Low-density lipoprotein receptor	100 (100–100)	0
	PCSK9	Proprotein convertase subtilisin/kexin type 9	100 (100–100)	0
Hypertrophic cardiomyopathy (HCM)	ACTC1	Actin, alpha, cardiac muscle 1	100 (100–100)	0
	CSRP3	Cysteine and glycine-rich protein 3 (cardiac LIM protein)	100 (100–100)	0
	MYBPC3	Myosin-binding protein C, cardiac	100 (100–100)	0
	MYH7	Myosin, heavy chain 7, cardiac muscle, beta	100 (99.9–100)	160
	MYL2	Myosin, light chain 2, regulatory, cardiac, slow	100 (100–100)	0
	MYL3	Myosin, light chain 3, alkali; ventricular, skeletal, slow	100 (100–100)	0
	TNNI3	Troponin I type 3 (cardiac)	100 (100–100)	0
	TNNT2	Troponin T type 2 (cardiac)	100 (100–100)	0
	TPM1	Tropomyosin 1 (alpha)	100 (100–100)	0
Long QT syndrome (LQTS)	KCNE1	Potassium voltage-gated channel, Isk-related family, member 1	100 (100–100)	0
	KCNE2	Potassium voltage-gated channel, Isk-related family, member 2	100 (100–100)	0
	KCNH2	Potassium voltage-gated channel, subfamily H (eag-related), member 2	100 (100–100)	0
	KCNJ2	Potassium inwardly rectifying channel, subfamily J, member 2	100 (100–100)	0
	KCNQ1	Potassium voltage-gated channel, KQT-like subfamily, member 1	100 (100–100)	0
	SCN5A	Sodium channel, voltage-gated, type V, alpha subunit	100 (100–100)	0
Noonan syndrome (NS)	KRAS	V-Ki-ras2 Kirsten rat sarcoma viral oncogene homolog	100 (100–100)	0
	PTPN11	Protein tyrosine phosphatase, non-receptor type 11	100 (100–100)	0
	RAF1	V-raf-1 murine leukemia viral oncogene homolog 1	100 (100–100)	0
	SOS1	Son of sevenless homolog 1 (Drosophila)	100 (100–100)	0
Phenocopy genes	GLA	Galactosidase, alpha	100 (100–100)	0
	LAMP2	Lysosomal-associated membrane protein 2	100 (100–100)	0
	PRKAG2	Protein kinase, AMP-activated, gamma 2 non-catalytic subunit	100 (100–100)	0

Genomic coordinates of regions with poor callability are given in Table S7

### Comparison Between Targeted ICC Sequencing, WES, Deep WES and WGS

**Fig. 1:**
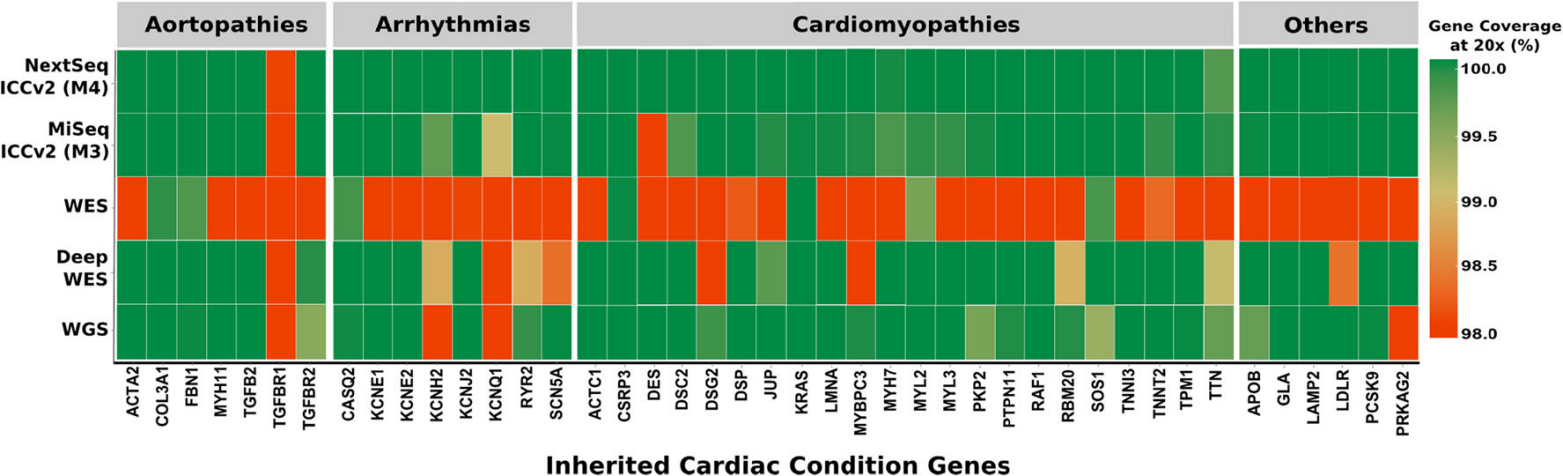
Stringent heat map showing the percentage coverage of ICC disease genes commonly used to inform clinical practice (*n* = 43) at 20× read depth using M3 (MiSeq, 150 bp PE), M4 (NextSeq 500, 150 bp PE), deep whole exome sequencing (WES; HiSeq 2500, 125 bp PE), WES (HiSeq, 125 bp PE) and whole genome sequencing (WGS: HiSeq X, 150 bp PE) (gene coverage at 20×: *dark red* ≤98 %; *dark green* = 100 %)

We then compared the performance of the final assay (ICCv2) against Nextera-based WES and WGS (Table 2; Fig. 1). Using routinely applied, off-the-shelf WES, most disease-causing ICC genes (*n* = 36/43) had suboptimal coverage (49–98 %, 20×). For a direct comparison at the same level of read depth as the ICC methods, deep WES (∼500×) was used. However, even with deep WES, ten disease genes remained poorly covered, an average of 208 bases had no coverage at all and the cost was extremely high ($5400). The performance of WGS at ∼75× average read depth was similar to deep WES at ∼520× read depth but at lower cost.

**Table 2:** Comparison of quality metrics for ICCv2 (marketed as the TruSight Cardio Sequencing Kit) panel (M3, MiSeq; M4, NextSeq 500), WES, Deep WES and WGS

Sequencing summary	Method 3 (M3)	Method 4 (M4)	WES	Deep WES	WGS
Nextera Rapid Capture kit	ICCv2	ICCv2	WES	WES	TruSeq Nano DNA
Sequencer	MiSeq	NextSeq 500	HiSeq 2500	HiSeq 2500	HiSeq X
Sequencing reagent kit	MiSeq v2, 300 cycles	Mid Output v2, 300 cycles	SBS v4, 250 cycles	SBS v4, 250 cycles	V2.5, 300 cycles
Samples per lane	12	48	12	2	0.5
Average output per sample (GB)	0.5	1.2	5.4	43.7	200
Mean read depth of ICC target (95 % CI)	329× (317×–342×)	578× (568×–587×)	74× (71×–78×)	522×	69.4× (65.4×–73.5×)
Mean ICC bases ≥20× (%) (95 % CI)	99.8 (99.8–99.9)	99.9 (99.9–99.9)	88.1 (87.3–88.9)	99.2	99.3 (99.2–99.5)
Targeted enrichment and sequencing cost per sample (USD)	200	200	900	5400	2800
Library preparation and sequencing time per run (days)	4	4	9	9	4

A full comparison of methods 1–4 using ICCv1 and ICCv2 panels is shown in Table S4

**Fig. 2:**
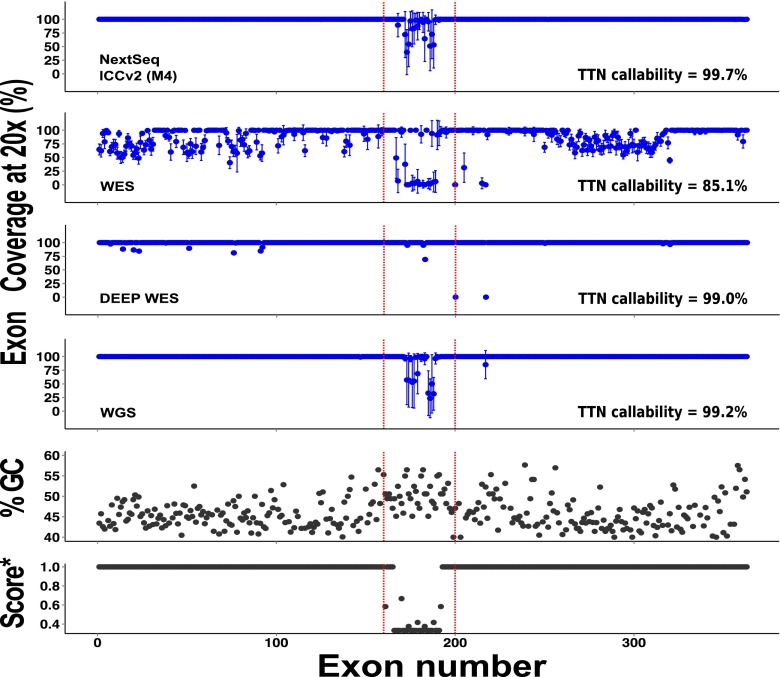
Percentage coverage of all TTN exons (ENST00000589042/NM_001267550.1) at 20× read depth across methods (*top four panels*). Mappability score (score* [28]) and GC content in the TTN gene (*bottom two panels*). *Error bars* represent standard deviation

By comparison, using the ICCv2 assay (M4, NextSeq), only three of the major ICC disease genes were not 100 % covered at 20× and an average of only 22 bases of target were not covered at all (Fig. 1, Table 1). Gene complexity was a major determinant of base coverage, especially for regions of high GC content and low mappability in the titin gene (Fig. 2) [29]. Perhaps surprisingly, mean read depth coverage of titin exons using deep WES was higher than M4 for regions of low complexity, perhaps reflecting greater library complexity for deep WES. However, the overall titin gene coverage at a mean read depth of 20× was best with our final assay on the NextSeq (99.7 %), less good with deep WES (99.0 %) and WGS (99.2 %) and poor with standard WES (85.1 %).

### Variant Calling Accuracy

Variant calling accuracy was assessed using an in-house pipeline using the reference NA12878 sample. Variants were assessed over a 522,763 bp region overlapping with the GIAB high confidence regions and which corresponded to the ICC target ±8 bps (to include essential splice site and proximal intronic regions). The ICC panel had very high accuracy as compared to GIAB benchmark calls dataset (Table 3). The MiSeq and NextSeq assays had near identical performance with SNV sensitivity of 100 %. The NextSeq data had one false positive that only just passed the minimum variant confidence/quality by depth (QD) threshold of 2.0 (QD = 2.1) and was of obvious low quality when viewed in IGV. The false negative indel missed by both platforms was an A deletion, 6 bp into an intron and before a run of 15 As. This variant was initially called on the MiSeq platform but filtered out due to low QD (chr12 22063251 CA C).

**Table 3:** Comparison of variant calls for M3 (MiSeq) and M4 (NextSeq) sequencing of the N12878 reference sample with the Genome in a Bottle high confidence variant call set

Sequencer	Variant type	TP	FP	FN	TN	Sensitivity (%)^a^	Precision (%)^b^	MCC (%)^c^
MiSeq	All	249	0	1	522509	99.6	100	99.8
MiSeq	SNVs	245	0	0	522518	100	100	100
MiSeq	Indels	4	0	1	522754	0.80	100	89.4
NextSeq	All	249	1	1	522508	99.6	99.6	99.6
NextSeq	SNVs	245	1	0	522517	100	99.6	99.8
NextSeq	Indels	4	0	1	522754	0.80	100	89.4

Analysis was done over a 522,763 bp region corresponding to protein-coding region ±8 bps that overlaps with the GIAB high confidence regions

*TP* true positive, *FP* false positive, *FN* false negative, *MCC* Matthews correlation coefficient

^a^Sensitivity = TP / (TP + FN)

^b^Precision = TP / (TP + FP)

^c^MCC = (TP × TN) − (FP × FN) / √[(TP + FP)(TP + FN)(TN + FP)(TN + FN)]

### Variant Calling Comparisons

Single nucleotide variant (SNV) calling was assessed using the DNA substitution rate, i.e. the ratio of transitions (Ts) to transversions (Tv). We observed a Ts/Tv ratio ∼3.5 across the targeted CDS region for our assays, concordant with previous findings [30]. A total of 65 samples from M3 (MiSeq, *n* = 23) and M4 (NextSeq 500, *n* = 42) were selected for variant calling comparisons using either our in-house pipeline (The GATK Best Practices workflow) or one of two Illumina BaseSpace Apps: Isaac enrichment v2.1 or BWA enrichment v2.1 (Table S5) [31]. For 65 samples, BaseSpace Apps completed jobs within 1 h as compared to locally run pipelines that took ∼2 h on a computational cluster (local cluster four CPU cores per job with 14 GB/CPU RAM allocation). We observed 98.8 % concordance between our in-house pipeline and both BaseSpace Apps for SNVs and indels with ∼100 % of SNVs detected locally also detected by both apps. A subset of 26 variants identified by our custom pipeline underwent Sanger sequencing and were all confirmed.

Variant calling assessment test (VCAT) [BaseSpace App, Illumina Inc.] was performed on variant call sets obtained from all three pipelines using reference sample NA12878 (*n* = 16, technical replicates). After comparing with gold standard GIAB high confidence calls v2.18 and Platinum genome v8.0, we observed 100 % precision for SNV and indel calls obtained from both the in-house, custom pipeline and BWA Enrichment app in BaseSpace. However, Isaac Enrichment variant call set had poorer performance with 97 % and below 50 % precision for SNV and indel calls respectively (Table S6).

## Discussion

In recent years, the use of targeted sequencing and WES for the study of ICCs has increased, reflecting high-throughput capabilities and reduced per-base costs of NGS when compared to conventional Sanger sequencing. Current NGS cardiac panels often represent a limited number of ICC genes (*n* = 9 to 88), and assay performance is variable, often requiring PCR-based gap filling and Sanger sequencing [15, 32–34]. The final ICC assay presented here includes 174 ICC genes that have primary, secondary or possible involvement in a wide range (>17) of ICCs (Table S1) including all 30 ACMG genes [11] and phenocopy genes [35]. However, common pathogenic variants that are outside the captured region cannot be assessed by this assay, for instance, the 25 bp deletion in intron 32 of MYBPC3 that has been associated with HCM [36].

The assay we describe represents over 85 genes implicated in cardiomyopathies including all major disease and phenocopy genes for HCM, DCM and ARVC [2, 37, 38]. Inherited arrhythmias are an important group of ICCs, and over 28 genes implicated in inherited arrhythmias including all major disease-causing genes for LQTS are included in the assay (Table 1, Table S1). The panel also includes over 14 genes implicated in inherited aortopathies, 12 of which are common to an established panel for thoracic aortic aneurysm [39]. The comprehensive nature of the panel we describe here makes it ideally suited for a single workflow in laboratories providing sequencing for multiple ICCs and for molecular pathology studies of sudden cardiac death [40, 41], although more specialised panels may offer advantages in focused/single disease laboratories. It is interesting to note that from a clinical point of view, the major disease-causing ICC genes used to inform clinical practise have not changed much over recent years. While the current panel is of fixed content, it would be possible to iterate the design in the future and to include intronic regions of interest, if the ICC community was to solicit this change.

It could, and has been, argued that simply using off-the-shelf WES is sufficient for ICC research and diagnostics [42]. However, the coverage of ICC genes using WES at manufacturer-recommended sequencing depths is insufficient for accurate variant calling for a number of ICC genes (Table 2, Fig. 1). Even with deep WES (∼500× read depth across ICC target), ICC gene coverage was less good than the optimised ICC-specific assay (Fig. 1). WGS may be better than WES for detecting exome variants [43], and we found the coverage of ICC genes to be good, but WGS comes with cost, incidental finding and data storage issues [44], and at an average of 70× coverage is not as good for ICC gene assessment as the assay we describe here.

It is important to consider differences in variant calling between informatic pipelines as highlighted by our comparison of three methods that use different mapping and variant calling algorithms and data pre-processing workflows. It has been reported that alignment with BWA-MEM and GATK HaplotypeCaller pipeline offers best sensitivity and precision [45]. Cloud-based and easily implemented pipelines on BaseSpace offer a viable alternative for those with limited in-house informatics and, based on preliminary analyses, have comparable sensitivity (Table S5). We suggest that individual users prioritise and use one pipeline and then work to identify pipeline-specific performance parameters. An advantage of using cloud-based processes is that computational hardware purchase and upkeep is not needed and the processing power accessible via BaseSpace is fast. Hence, small laboratories can readily access both processing power and informatics tools.

In summary, the ICC gene panel described here provides high and uniform coverage (99.9 % targeted region at >20×), ‘clinical-grade’ sequencing with up to 100 % sensitivity and precision for SNVs and indels in the protein-coding regions of ICC genes. This raises the question as to whether or not ‘Sanger validation’ is required as part of a clinical workflow; the data presented here would suggest not. As compared to the WES, deep WES and WGS, this assay has better performance, shorter turnaround times, lesser informatics requirements and lower sequencing costs. While assessment of structural variation remains a challenge, the very deep coverage this panel affords may provide ways to interrogate this in the future. We believe that this panel will be important for ICC research and ultimately clinical genetic investigation of ICCs and for molecular autopsy. This panel is now available commercially (TruSight Cardio Sequencing Kit; research use only), and with the ease of use of cloud-based computational processing and informatics, it is widely accessible for users.

## Electronic supplementary material

ESM 1 (XLSX 199 kb)
